# Hepatobiliary Adverse Reactions during Treatment with Cladribine: Analysis of Data from the European Spontaneous Reporting System

**DOI:** 10.3390/ph16081071

**Published:** 2023-07-27

**Authors:** Elena Mirabela Velișcu, Valerio Liguori, Antonietta Anatriello, Giorgia Teresa Maniscalco, Andrea Cantone, Luigi Di Costanzo, Pasquale Stefanelli, Cristina Scavone, Annalisa Capuano

**Affiliations:** 1 Eu2P Programme, University Bordeaux, 146, rue Léo Saignat, 33076 Bordeaux, France; mirabela_milea@yahoo.co.uk; 2 Department of Experimental Medicine, University of Campania “Luigi Vanvitelli”, 80138 Naples, Italy; valerio.liguori@unicampania.it (V.L.); andreacantone9800@gmail.com (A.C.); luigi.dicostanzo@studenti.unicampania.it (L.D.C.); annalisa.capuano@unicampania.it (A.C.); 3 Regional Center of Pharmacovigilance and Pharmacoepidemiology of Campania Region, 80138 Naples, Italy; 4 Multiple Sclerosis Regional Center, “A. Cardarelli” Hospital, 80131 Naples, Italy; 5 Neurological Clinic and Stroke Unit, “A. Cardarelli” Hospital, 80131 Naples, Italy; 6 Dipartimento Farmaceutico, UOC Farmaceutica Convenzionata e Territoriale, ASL Napoli 1 Centro, 80131 Naples, Italy; pasqualestefanelli@gmail.com

**Keywords:** adverse drug reactions, cladribine, EudraVigilance, hepatic safety, multiple sclerosis

## Abstract

Background. Cladribine belongs to the group of disease-modifying therapies (DMTs) used to treat multiple sclerosis (MS). According to the highlights of a meeting held by the Pharmacovigilance Risk Assessment Committee (PRAC) on 14 January 2022, cladribine may be associated with the occurrence of liver injury, and thus liver function monitoring is recommended. Objectives and methods. Using data from the European spontaneous reporting database (EudraVigilance-EV), we aimed to describe the main characteristics of Individual Case Safety Reports (ICSRs) reporting cases of hepatobiliary disorders related to cladribine. The reporting odds ratio (ROR) was calculated to provide the probability of reporting hepatobiliary ICSRs among DMTs used to treat MS. Results. Overall, 118 ICSRs described the occurrence of cladribine-induced hepatobiliary ADRs. The majority of the ICSRs reported ADRs that were classified as serious (93%), and the outcome was mostly reported as “unknown” (50.8%). The most reported hepatobiliary disorders were drug-induced liver injury, abnormal hepatic function, ALT increases, liver disorders, hepatic failure, jaundice, lymphocyte count decreases, hepatotoxicity and hypertransaminasemia. The majority of cladribine-induced hepatic ADRs occurred in female patients belonging to the age group of 18–65 years. Conclusion. Considering the seriousness of cladribine-induced hepatic ADRs, a close monitoring of patients receiving this drug is highly recommended. In this context, further pharmacovigilance studies evaluating the hepatic safety profile of cladribine are strongly needed.

## 1. Introduction

Multiple sclerosis (MS) is the most widespread autoimmune disease of the central nervous system (CNS) and affects almost 2 million people globally. MS is an inflammatory neurodegenerative disease and has been demonstrated to be significantly influenced by genetic and environmental factors, obesity, vitamin D levels, Epstein-Barr viral infections and smoking [1,2].

Among the disease-modifying therapies (DMTs) authorized for the treatment of MS, cladribine was approved in 2017 [3]. The drug is approved for the treatment of relapsing multiple sclerosis (RMS), secondary progressive MS (SPMS) and relapsing–remitting multiple sclerosis (RRMS) in adult patients [4,5]. Cladribine is an anti-pyrimidine and anti-metabolite agent [6] that is biologically active in selected cell types. The drug is able to sustain and target the reduction in circulating B and T lymphocytes, which in MS activates a cascade of inflammatory cytokines and antibodies directed against various CNS components [6,7]. Thus, cladribine reduces these immune responses by targeting active immunity.

According to data from the 96-week phase III trial CLARITY [8], 90% of the patients treated with cladribine completed treatment, with rare discontinuations as a result of adverse events (AEs). Similar findings were observed in the CLARITY extension trial, in which lymphopenia (mainly mild or moderate) was the most commonly reported AE and reason for treatment discontinuation [9]. The results of a post-hoc analysis of CLARITY and ORACLE revealed that the most common AEs reported with cladribine tablets were headache, lymphopenia and nausea [10].

On 14 January 2022, the European Medicines Agency (EMA) published on its website the meeting highlights from the Pharmacovigilance Risk Assessment Committee (PRAC) from 10 to 13 January 2022 [11]. The EMA aimed to inform healthcare professionals about the AEs of liver injury with cladribine therapy and provided new recommendations for liver function monitoring.

Considering the clinical significance of cladribine-related liver injury, the present study aimed to evaluate the occurrence of these events during treatment with cladribine by describing data from Individual Case Safety Reports (ICSRs) retrieved from the European spontaneous reporting system (EudraVigilance-EV). We also aimed to estimate the reporting probability of hepatobiliary disorders among the DMTs used to treat MS.

## 2. Results

As of May 2023, 4181 ICSRs reporting cladribine as a suspected drug had been received by the EV database. As shown in Table 1, 118 ICSRs, covering 562 PTs, reported ADRs belonging to “hepatobiliary disorders” according to the Medical Dictionary for Regulatory Activities (MedDRA^®^) System Organ Classes (SOCs). The majority of patients who experienced hepatobiliary ADRs belonged to the age group of 18–64 years (68.6%), and a higher proportion of them were female (61.0%). ICSRs were mainly reported by HCPs, while no substantial difference was found in terms of the primary source country for regulatory purposes. Apart from eight ICSRs in which the seriousness degree was not defined, the remaining one hundred and ten ICSRs described hepatic ADRs that were classified as serious (mainly as “Other Medically Important Condition” and “Caused/prolonged hospitalization”). Regarding outcomes, 60 ICSRs did not report any information, while 16 ICSRs reported a fatal outcome (Table 2).

The majority of the ICSRs reported cladribine as the only suspected drug (n = 70). The most reported suspected drugs other than cladribine were cytarabine (nine cases), followed by cyclophosphamide (six cases), rituximab (six cases), methylprednisolone (five cases), mitoxantrone (five cases) and prednisolone (three cases). Lastly, concomitant medications were reported in 56 ICSRs (Table 1).

As reported in Table 3, the following PTs were reported in more than 10 ICSRs: “Drug-induced liver injury”, “Hyperbilirubinaemia”, “Pyrexia”, “Hepatic function abnormal”, “Pancytopenia”, “Alanine aminotransferase increased” and “Liver disorder”.

As reported in Table 4, cladribine had a higher reporting probability of ICSRs with ADRs belonging to “Hepatobiliary disorders” according to SOCs compared with glatiramer acetate, natalizumab, ofatumumab, peginterferon beta 1a, ocrelizumab, dymethil fumarate and dyroximel fumarate (with RORs equal to 2.06 (1.66–2.55), 1.46 (1.20–1.79), 2.22 (1.47–3.34), 1.56 (1.20–2.03), 2.66 (2.03–3.48), 1.61 (1.31–1.97) and 2.29 (2.26–4.17), respectively, all of which are statistically significant).

## 3. Discussion

Using data from the EV database, we analysed the safety profile of cladribine in terms of hepatobiliary ADRs. Our choice to evaluate this specific concern was based on a recent statement released by the EMA in which the agency highlighted the risk of hepatobiliary adverse events associated with this drug. In addition, considering that drug-induced hepatic toxicities can range from asymptomatic mild elevation in hepatic enzymes to liver failure and death, the monitoring of DILI is essential during treatment with hepatotoxic drugs, and consequently the assessment of the risk of DILI in newly authorized drugs should represent a priority for worldwide pharmacovigilance systems.

We found 118 ICSRs describing cases of hepatobiliary disorders associated with cladribine. Hepatic ADRs mainly occurred in female patients and patients belonging to the age group of 18–65 years. Both of these datasets are expected if we consider that cladribine is used to treat MS, which is more common in women and adults in general [12] (the prevalence ratio of MS in women to men is 2.3–3.5:1). In addition, women tend to experience all types of ADRs more commonly than men, due to hormonal and biological factors [13,14,15].

The hepatic ADRs were mainly classified as serious, including 16 ICSRs that reported fatal cases. In line with our data, some case reports described the occurrence of serious and fatal hepatic ADRs during treatment with a DMT. For instance, a case series described the occurrence of severe hepatitis in two patients, a 41-year-old woman and a 29-year-old woman, who were receiving glatiramer acetate [16]. Another case report described the occurrence of a fatal hepatitis in a 44-year-old woman with MS who was receiving IFN-beta-1a treatment and was admitted to a local hospital for severe icterus and liver injury [17]. Lastly, other studies have reported the occurrence of fulminant hepatitis during treatment with ocrelizumab and IFN-β plus steroids in MS patients, mainly women [18,19].

The higher rate of ICSRs reported by HCPs matches with that of the results from other studies that have analysed ICSRs from national spontaneous reporting systems or from the EV [20,21,22,23,24,25,26].

We found that the most common reported PTs were “Drug-induced liver injury” (DILI), “Hyperbilirubinaemia”, “Hepatic function abnormal”, “Alanine aminotransferase increased”, “Liver disorder” and “Hepatic failure”. DILI can be a serious predictable or unpredictable consequence of many pharmacological treatments. Its occurrence depends on the genetic and environmental risk factors that are responsible for the modification of hepatic metabolism and the excretion of drugs, which in turn lead to cellular stress, cell death and the activation of an adaptive immune response that progresses to liver injury. Although rare, idiosyncratic DILI can be extremely serious and, in some cases, fatal [27,28]. Based on its features, DILI is constantly receiving attention from pharmaceutical companies, regulators, researchers and clinicians. Indeed, it commonly represents the most frequent cause for stopping drug development or restricting indications after marketing authorization [29]. Literature data have revealed a case report describing the occurrence of DILI in a patient receiving cladribine [30]. The case report concerned a 19-year-old female patient with a history of fatty liver but normal liver function tests who had received high-dose corticosteroids and cladribine. The patient received treatment with 70 mg cladribine in week 1; however, after 12 days she developed emesis, jaundice, nausea, itching, headache and an erythematous skin reaction. Therefore, she received treatment with cetirizine and 25 mg prednisolone tablets daily for 6 days. Her blood tests 5 days after the symptoms relapsed indicated acute liver injury. The blood tests detected grade 4 elevation of ALT and grade 3 elevation of total bilirubin and AST. After 9 days, a mild coagulopathy was detected as well. The authors concluded that that the association between the steroids and liver injury was not considered probable as the tests were normal up to 6 weeks after the patient was treated with methylprednisolone, while the association between cladribine and liver injury was considered probable as the tests indicated abnormal values 18 days after the last treatment tablet of cladribine. Furthermore, the results of the diagnostic algorithm RUCAM (Roussel Uclaf Causality Assessment Method) indicated a probable association between cladribine and liver injury. In phase III clinical trials, liver enzyme abnormalities were uncommon and elevated transaminases >5 ULN were reported in less than 2% of the subjects. Post-marketing data did not reveal a high risk of hepatotoxicity. However, very few cases of HBV reactivation have been registered [8,31,32,33,34]. Leist and al. reported several integrated analyses regarding the safety of cladribine during the clinical development program for MS. The data analysed in July 2022 concerned 56,300 subjects who received cladribine and 95,664 patient-years of exposure since its approval in 2017. The results concluded that liver AEs (generally enzyme elevations) were not commonly recorded in temporal association with cladribine. The majority of liver injury cases were classified as grade 1 (mild, 43 reports) or 2 (moderate, 14 reports); there were also one grade 4 case (fatal, which was associated with isoniazid toxicity) and two grade 3 cases (severe). The majority of liver AEs occurred within 8 weeks of initiating the first course of treatment with cladribine. The authors reported that, although uncommon, liver toxicity represents an important risk associated with cladribine. Therefore, further guidance on monitoring liver function was determined as a part of the modified prescription information [35].

Apart from DILI, eleven cases of increased alanine aminotransferase (ALT) and seven cases of aspartate aminotransferase (AST) were also found in the retrieved ICSRs. In line with this finding, the CLARITY and ORACLE studies reported increases in ALT more frequently than increases in AST. Moreover, in the literature, there are mentioned cases of MS patients treated with cladribine who have increased GGT (gamma-glutamyl transferase) after treatment [5]. However, in phase III trials, it was noticed that abnormal liver function tests were uncommon. Furthermore, grade 3 transaminase elevation was reported in less than 2 percent of the patients [8,31,36]. Indeed, in the CLARITY study, out of 884 patients exposed to cladribine over 2 years, less than 10% and 2% experienced grade 1 and 3 AST/ALT elevation, respectively [8]. On the other hand, in the ORACLE study, out of 616 patients exposed to cladribine over 2 years, less than 5% and 1% experienced grade 1 and grade 3 AST/ALT elevation, respectively. Similar findings were reported by the ONWARD study [36].

Among the ICSRs retrieved for this study, apart from the PTs related to liver disorders, we also found a few cases of hyperbilirubinaemia, pyrexia, pancytopenia and lymphocytopenia. In line with this finding, it is known that neutropenic fever is the most common complication of cladribine therapy for hairy-cell leukemia (HCL), leading to a 3% mortality rate [37]. In addition, as reported by Leist et al. [33], due to its mechanism of action, cladribine leads to a transient and selective lymphocyte reduction; thus, lymphopenia is an expected adverse event. Data from the CLARITY extension and PREMIERE registry studies concluded that cladribine can lead to a reduction in lymphocyte B and lymphocyte T [38]. The CLARITY study reported lymphocytopenia mainly graded as moderate or mild, which was more frequently reported in patients who received cladribine compared to those receiving a placebo [8]. The literature has also concluded that herpes zoster is occurring more frequently during periods of severe lymphopenia compared to periods without severe lymphopenia [8,9,39]. This will be further discussed, as the CLARITY study showed that there is a low risk of severe lymphopenia or clinical worsening after treatment with cladribine [9]. There is also a high rate of lymphopenia being reported as an adverse drug reaction for cladribine, perhaps due to the mechanism of action in clinical trials. In the CLARITY study, grade 3 or 4 lymphopenia cases have been reported in 25.6% of the patients receiving cladribine tablets compared to 0.5% of the patients who received a placebo [40]. Moreover, Rolfes et al. reported that lymphopenia was the most commonly observed AE (86.3% of patients; grade III–IV lymphopenia: 38.8%) [41].

Regarding the ROR among DMTs, we found a higher reporting probability of hepatic toxicity with cladribine compared with that of glatiramer acetate, natalizumab, ofatumumab, peginterferon beta 1a, ocrelizumab, dymethil fumarate and dyroximel fumarate. As reported by Biolato [4], none of the DMTs currently available for the treatment of MS are free of potential hepatic toxic effects. Indeed, cases of liver injury have been reported for beta-interferon, fingolimod, natalizumab, alemtuzumab and ocrelizumab. The mechanisms underlying the occurrence of DMT-induced DILI and hepatic ADRs in general are varied, ranging from autoimmune disorders (such as in cases associated with beta-interferon) or viral reactivation (such as fingolimod- or natalizumab-induced cases). A recent narrative review showed that beta-interferon is associated with an increase in AST levels [4]. Similarly, phase III studies have shown that up to 50% of patients have abnormal liver function tests linked to the use of dimethyl fumarate [42,43,44]. Safety data from another study showed that 1–2% of patients discontinued the treatment with dimethyl fumarate because of hepatic side effects [45]. Concerning alemtuzumab, data from the literature suggest that a small number of enrolled patients had abnormal liver function in a phase III trial, <1% of which was rated as serious [4,46,47].

Antonazzo et al. carried out an analysis over a 13-year period about DILI events related to both DMTs and symptomatic therapy (fampridine) by using data from the FDA Adverse Event Reporting System (FAERS). DILI events were divided into 8982 overall liver injuries (OLIs) and 4873 severe liver injuries (SLIs). In line with our study, most of these events happened in females (75%) >30 years old (72%). The results of the disproportionate reporting analysis revealed a higher hepatotoxic potential damage with interferons and mitoxantrone. In addition, the study also reported DILI events for more recently developed DMTs, such as alemtuzumab, teriflunomide and fingolimod [48]. Furthermore, another study, carried out by Francis et al., evaluated patients’ liver function after interferon-β-1a therapy, both in clinical trials and for post-marketing monitoring. They used a clinical trial database containing information about six studies which reported the use of interferon-β-1a in patients with MS for at least 6 months: five were placebo-controlled and another one involved a different formulation of interferon-β-1a. In the treated patients, considerable increases in ALT levels, asymptomatic cases, dose-related cases and all grades of severity were indicated. More than 50% of the elevations in liver enzymes were flagged during the first 3 months of treatment, and more than 75% occurred during the first 6 months. In some cases, a dosage reduction was required; in other cases, elevated enzyme levels would resolve spontaneously. Only 0.4% of the patients interrupted the interferon-β-1a treatment due to hepatic adverse effects. Concomitant medication use was not associated with an increased risk [49].

Lastly, as recently reported by Meunier and Larrey [50], the occurrence of DILI depends also on genetic factors, including the presence of polymorphisms of HLA which are associated with a higher susceptibility to idiopathic autoimmune hepatitis and liver injury induced by MS therapies. In addition, in our study, the role of suspected drugs other than cladribine as well as the role of concomitant medications and MS itself in the occurrence of liver toxicities cannot be excluded. Indeed, the prevalence of autoimmune hepatitis is reported to be higher among MS patients (0.17%) than in the general population (0.017%) [51].

In conclusion, considering the seriousness of cladribine-induced hepatic ADRs (in our study, almost 90% of the ICSRs described serious ADRs), a close monitoring of patients receiving this drug is highly recommended. As recently reported by Clavelou P. et al. [52], although uncommon, cladribine-induced liver disease might represent a serious threat for patients; thus, it is important to withhold cladribine treatment from people with pre-existing liver disease and stay vigilant in regards to the emergence of liver disease during treatment. According to EMA recommendations, HCPs are advised to perform a detailed review of patient history of underlying liver disorders or incidence of liver injury with other medicines before treatment with cladribine is started [53]. In all patients eligible for cladribine treatment, liver function tests, including serum aminotransferase, alkaline phosphatase and total bilirubin levels, should be assessed prior to the initiation of therapy in years 1 and 2. In addition, during treatment, liver function tests should be conducted and repeated as necessary. If a patient develops liver injury, treatment with cladribine should be interrupted or discontinued, as appropriate.

## 4. Methods

### 4.1. Data Source

ICSRs reporting cases of cladribine-induced hepatobiliary disorders were retrieved from the EV database, which is publicly accessible at https://www.adrreports.eu/en/search.html (accessed on 20 June 2023). Data used for this analysis pertained ICSRs related to cladribine that were uploaded in the EV database before and up to May 2023. EV is the European pharmacovigilance database that collects adverse reactions related to drugs and vaccines that are authorized in the European Economic Area (EEA) [54]. The database is managed by the EMA on behalf of the EU drug regulatory network.

### 4.2. Selection of Individual Case Safety Reports with Line Listing

All ICSRs that reported at least one hepatobiliary disorder as ADR having cladribine as a suspect drug (reported with the substance name) were analysed using the line listing tool from the EV. To recognize ICSRs that reported hepatobiliary disorders, the Medical Dictionary for Regulatory Activities (MedDRA^®^) System Organ Classes (SOCs) of “hepatobiliary disorders” was used.

### 4.3. Data Analyses

All data provided in each ICSR regarding patient characteristics (age group and gender), hepatobiliary ADR (preferred term (PT), seriousness and outcome), suspected drug(s) other than cladribine, number of concomitant medications, primary source country for regulatory purpose and reporter type (HCP or non-HCP) were analysed.

A case was classified as “serious” when the ADR led to death, when it was life-threatening or required hospitalization or prolongation of existing hospitalization, when it resulted in significant or persistent incapacity/disability, when it was a congenital anomaly/birth defect or when it was classified as some other medically important conditions [55]. The outcome was classified as “recovering/resolving”, “recovered/resolved”, “recovered/resolved with sequelae”, “not recovered/not resolved”, “fatal” or “unknown”. In cases in which more than one ADR was reported related to the Standardized MedDRA Query (SMQ) or the same SOC, but different outcomes were reported, the outcome with the lower level of resolution was selected to perform the classification.

We also aimed to assess the reporting odds ratio (ROR) and 95% confidence interval (CI), as well as the Chi-square test, to evaluate if cladribine has a lower/higher probability of reporting ICSRs with hepatobiliary ADRs compared with the other DMTs used to treat MS.

### 4.4. Ethical Standards

Safety data extracted from the spontaneous reporting system comply with ethical standards and are anonymous. Therefore, no further ethical measures were required.

## 5. Strengths and Limitations

To our knowledge, this is the first study that has evaluated the hepatic safety profile of cladribine using data from the EV database. The data we have analysed is of high importance considering that they refer to ICSRs collected during routine clinical practice, when drugs are used in real-life conditions. Indeed, although randomized controlled trials represent the gold standard for determining the efficacy and safety of treatments, their intrinsic limitations undermine the results’ generalizability. On the contrary, data collected in real-life conditions reflect the heterogeneity of people using drugs, such as patients with comorbid conditions, those taking multiple medications, geriatric patients and other vulnerable populations who are normally excluded by clinical trials, and thus are useful. Therefore, this study represents a comprehensive analysis of hepatobiliary ADRs’ safety data and has importance and value in characterizing the safety profile of cladribine using data from the EV databases.

Although spontaneous reporting systems are valuable due to their strengths, they have also limitations, such as the low reporting rate of ADRs or incomplete or inaccurate information. Underreporting leads to economic, ethical and health burdens. HCPs are usually the primary reporters for ADRs, and they represent a great source for collecting post-marketing ICRSs [21]. This unreported information (e.g., outcome, medical history, seriousness, concomitant medication and further suspected drugs) has a high importance and might have impacted this analysis. In addition, analysing data from ICSRs present in the EV database is not always the proper method to assess tolerability issues. In fact, aggregate data available through the public access online tools are mainly useful for descriptive analyses, but their interpretation is difficult from a clinical point of view. Lastly, the limitations of this study are the same as other studies based on data from the spontaneous reporting system, namely the poor quality of data and the lack of data on drug exposure.

## 6. Conclusions

We carried out a descriptive analysis of ICSRs reporting hepatobiliary disorders associated with cladribine based on the data from the EV database. The majority of the retrieved ICSRs reported ADRs that were classified as serious and occurred in female patients in the age group of 18–64 years. The majority of ICSRs reported cladribine as the only suspected drug, and the most commonly reported PTs were “Drug-induced liver injury”, “Hyperbilirubinaemia”, “Pyrexia”, “Hepatic function abnormal”, “Pancytopenia”, “Alanine aminotransferase increased” and “Liver disorder”.

Based on the data retrieved from the EV and considering that the majority of the ICSRs described serious hepatic ADRs, we believe that further pharmacovigilance studies evaluating the hepatic safety profile of cladribine are needed. Post-marketing adverse event reporting is of high importance in order to allow for the early recognition and treatment of ADRs. Moreover, assessments of the physiopathological mechanism underlying drug-induced hepatic toxicity supply clinicians with valuable instruments for prevention and treatment.

## Figures and Tables

**Table 1:** Demographic and clinical characteristics of ICSRs reporting ADRs related to “hepatobiliary disorders” according to SOCs with cladribine as suspected drug and retrieved from the EudraVigilance databases from January 2017 to May 2023.

Variable	Level	All ICSRs N = 118 (100%)
Age	2 months–2 years	4 (3.4)
	3–11 years	2 (1.7)
	18–64 years	81 (68.6)
	65–85	14 (11.9)
	Not specified	17 (14.4)
Sex	Female	72 (61.0)
	Male	42 (35.6)
	Not specified	4 (3.4)
Primary source qualification	Healthcare professional	114 (96.6)
	Non-healthcare professional	4 (3.4)
Primary source country for regulatory purposes	European Economic Area	62 (53.0)
	Non-European Economic Area	56 (47.0)
Seriousness degree	Caused/prolonged hospitalization	37 (31.4)
	Life-threatening	8 (6.8)
	Other Medically Important Condition *	50 (42.3)
	Results in death	15 (12.7)
	Unknown	8 (6.8)
Outcome	Unknown	60 (50.8)
	Recovering/Resolving	14 (11.9)
	Recovered/Resolved	15 (12.7)
	Not recovered/Not resolved	12 (10.2)
	Not specified	1(0.9)
	Fatal	16 (13.5)
Suspected drug other than cladribine	0	70 (59.3)
	1	21 (17.8)
	2	6 (5.1)
	3	7 (5.9)
	4	4 (3.4)
	≥5	10 (8.5)
Concomitant drugs	1	18 (15.3)
	2	5 (4.2)
	3	8 (6.8)
	4	4 (3.4)
	≥5	21 (17.8)
	Not reported	62 (52.5)

* One of these ICSRs reported ADRs with a fatal outcome.

**Table 2:** ICSRs with cladribine as suspected drug and cases with a fatal outcome.

Case No.	Age Group	Sex	PT	Suspect Drug(s) Other than Cladribine	Concomitant Medication
1	65–85 Years	F	Acute kidney injury, acute respiratory failure, alcoholic liver disease, drug-induced liver injury, hepatic encephalopathy, lactic acidosis, lymphocyte count decreased, rash, tuberculosis	Isoniazid	Fampridine, alendronic acid, estradiol, glyceryl trinitrate, metoprolol succinate, metoprolol tartrate, naproxen, omeprazole, simvastatin, tizanidine, valaciclovir
2	3–11 Years	F	Acute graft versus host disease, cholestasis, diarrhoea, lymphopenia, multiple organ dysfunction syndrome, off-label use, pyrexia, rash	-	Ciclosporin
3	18–64 Years	M	Febrile neutropenia, generalized oedema, liver injury, non-cirrhotic portal hypertension, renal injury, varices oesophageal	-	-
4	18–64 Years	M	Aspergillus infection, chest pain, ineffective drug, dyspnoea, erythropoiesis abnormal, hairy cell leukaemia, hepatomegaly, leukopenia, lymphocytosis, neutropenia, pulmonary thrombosis, pyrexia, respiratory failure, respiratory tract infection (fungal), splenic infarction, splenic infection (fungal), splenomegaly	Filgrastim, rituximab, amphotericin b	Vemurafenib
5	18–64 Years	F	Hepatic failure, hepatitis fulminant	Rituximab	Lamivudine, diclofenac, famotidine/omeprazole, glutathione, glyceryl phosphate, mequitazine, prednisolone, sulfamethoxazole/trimethoprim, ursodeoxycholic acid
6	18–64 Years	M	Febrile neutropenia, hepatic failure, hyponatraemia, pulmonary embolism	Alemtuzumab, cyclophosphamide, doxorubicin, etoposide, pentostatin, prednisone, vincristine	-
7	3–11 Years	M	Acute myeloid leukaemia, aspergillus infection, disease progression, febrile neutropenia, systemic mycosis, incomplete therapeutic product effect, toxicity to various agents, venoocclusive liver disease	Topotecan	Filgrastim
8	65–85 Years	F	Cerebral infarction, herpes zoster, hyperbilirubinemia, myelosuppression, pancytopenia, sepsis	-	Diltiazem hydrochloride, metoprolol tartrate
9	18–64 Years	F	Cardiac failure congestive, hepatotoxicity, multiple organ dysfunction syndrome, neurotoxicity, sepsis	Anti-T-lymphocyte immunoglobulin for human use/rabbit, busulfan	-
10	18–64 Years	F	State of confusion, graft versus host disease, hepatitis, pancytopenia, pyrexia, rash	-	2-aminopyridine
11	65–85 Years	M	Infection, liver disorder, mantle cell lymphoma refractory, nephropathy, pancytopenia	Rituximab, cyclophosphamide, doxorubicin, vincristine sulfate	Cytarabine, filgrastim, methotrexate, cefepime hydrochloride, famotidine, pazufloxacin mesilate, platelet concentrate, prednisolone sodium succinate, red blood cells, vancomycin
12	65–85 Years	F	Hepatic failure, oedema, renal failure, T-cell chronic lymphocytic leukaemia	-	-
13	18–64 Years	M	Deep vein thrombosis, enteritis, graft versus host disease, hepatic failure, myelosuppression, renal disorder, renal failure, septic shock	Ciclosporin, busulfan	Filgrastim, aciclovir, cefoperazone sodium/sulbactam sodium, ciprofloxacin hydrochloride, fluconazole, platelet concentrate, red blood cells, sulfamethoxazole/trimethoprim
14	18–64 Years	F	Abdominal pain (upper), alanine aminotransferase increases, aspartate aminotransferase increases, blood lactate dehydrogenase increases, chills, hepatic necrosis, herpes simplex, lymphocytosis, neutropenia	-	-
15	18–64 Years	M	Adenovirus infection, anaemia, blood albumin decreased, blood urea increased, liver injury, neutrophil count decreases, platelet count decreases, protein total decreases, ureteric stenosis, white blood cell count decreases	Rituximab, cyclophosphamide	Filgrastim, prednisone
16	18–64 Years	M	Depressed level of consciousness, fatigue, hepatitis fulminant, hyperbilirubinemia, hypertransaminasemia, lymphopenia, malaise, mucormycosis, paralysis, systemic mycosis	-	Chemotherapy (not specified), levofloxacin, amoxicillin/clavulanic acid, amphotericin b deoxycholate, azithromycin, cefotaxime, ciprofloxacin, piperacillin

**Table 3:** Distribution of ICSRs having cladribine as suspected drug by preferred terms (hepatobiliary disorders and non-hepatobiliary disorders).

List of PTs	N (%)
Drug-induced liver injury	14 (2.5)
Hyperbilirubinemia	13 (2.3)
Pyrexia	13 (2.3)
Hepatic function abnormal	12 (2.1)
Pancytopenia	12 (2.1)
Alanine aminotransferase increased	11 (2.0)
Liver disorder	11 (2.0)
Hepatic failure	9 (1.6)
Jaundice	9 (1.6)
Lymphocyte count decreased	9 (1.6)
Hepatotoxicity	8 (1.4)
Hypertransaminasemia	8 (1.4)
Neutropenia	8 (1.4)
Rash	8 (1.4)
Aspartate aminotransferase increased	7 (1.2)
Gamma-glutamyltransferase increased	7 (1.2)
Hepatic steatosis	6 (1.1)
Hepatitis	6 (1.1)
Hepatomegaly	6 (1.1)
Lymphopenia	6 (1.1)
Myelosuppression	6 (1.1)
Protein total decreased	6 (1.1)
Renal failure	6 (1.1)
White blood cell count decreased	6 (1.1)
Cholelithiasis	5 (0.9)
Cholestasis	5 (0.9)
C-reactive protein increased	5 (0.9)
Dyspnoea	5 (0.9)
Fatigue	5 (0.9)
Febrile neutropenia	5 (0.9)
Liver injury	5 (0.9)
Platelet count decreased	5 (0.9)
Anaemia	4 (0.7)
Decreased appetite	4 (0.7)
Infection	4 (0.7)
Multiple sclerosis relapse	4 (0.7)
Nausea	4 (0.7)
Off-label use	4 (0.7)
Thrombocytopenia	4 (0.7)
Transaminases increased	4 (0.7)
Blood alkaline phosphatase increased	3 (0.5)
Blood bilirubin increased	3 (0.5)
Blood lactate dehydrogenase increased	3 (0.5)
Diarrhoea	3 (0.5)
Hepatitis toxic	3 (0.5)
Hypersplenism	3 (0.5)
Hypertension	3 (0.5)
Hypotension	3 (0.5)
Hypothyroidism	3 (0.5)
Product use in unapproved indication	3 (0.5)
Renal impairment	3 (0.5)
Splenomegaly	3 (0.5)
Other PTs	247 (44.0)
Total PTs	562 (100.0)

**Table 4:** Reporting odds ratio of ICSRs with ADRs belonging to the SOCs’ ‘Hepatobiliary disorders’ for the comparison of cladribine vs. DMTs.

DMT under Evaluation	Other DMTs	ROR	*p*-Value
cladribine	interferon beta 1a	0.95 (0.78−1.15)	0.62
cladribine	interferon beta 1b	0.94 (0.76−1.16)	0.58
cladribine	glatiramer acetate	2.06 (1.66−2.55)	<0.001
cladribine	natalizumab	1.46 (1.20−1.79)	<0.001
cladribine	alemtuzumab	1.08 (0.87−1.34)	0.46
cladribine	teriflunomide	1.12 (0.91−1.37)	0.26
cladribine	ofatumumab	2.22 (1.47−3.34)	<0.001
cladribine	peginterferon beta 1a	1.56 (1.20−2.03)	<0.001
cladribine	fingolimod	1.01 (0.82−1.22)	0.93
cladribine	siponimod	0.99 (0.70−1.41)	0.99
cladribine	ozanimod	1.82 (1.00−3.30)	0.045
cladribine	ocrelizumab	2.66 (2.03−3.48)	<0.001
cladribine	dymethil fumarate	1.61 (1.31−1.97)	<0.001
cladribine	dyroximel fumarate	2.29 (2.26−4.17)	<0.05

## Data Availability

Data analysed for this article were retrieved from the EV database that is publicly accessible.

## References

[1] Ascherio A., Munger K.L. (2016). Epidemiology of Multiple Sclerosis: From Risk Factors to Prevention—An Update. Semin. Neurol..

[2] Reich D.S., Lucchinetti C.F., Calabresi P.A. (2018). Multiple Sclerosis. N. Engl. J. Med..

[3] EMA—Mavenclad—Summary of Product Characteristics. https://www.ema.europa.eu/en/documents/product-information/mavenclad-epar-product-information_en.pdf.

[4] Biolato M., Bianco A., Lucchini M., Gasbarrini A., Mirabella M., Grieco A. (2021). The Disease-Modifying Therapies of Relapsing-Remitting Multiple Sclerosis and Liver Injury: A Narrative Review. CNS Drugs.

[5] Schönfelder K., Schuh H., Pfister F., Krämer J., Eisenberger U., Skuljec J., Hackert J., Ruck T., Pfeuffer S., Fleischer M. (2021). Autoimmune glomerulonephritis in a multiple sclerosis patient after cladribine treatment. Mult. Scler..

[6] Janiec K., Wajgt A., Kondera-Anasz Z. (2001). Effect of immunosuppressive cladribine treatment on serum leucocytes system in two-year clinical trial in patients with chronic progressive multiple sclerosis. Med. Sci. Monit. Int. Med. J. Exp. Clin. Res..

[7] Niezgoda A., Losy J., Mehta P.D. (2001). Effect of cladribine treatment on beta-2 microglobulin and soluble intercellular adhesion molecule 1 (ICAM-1) in patients with multiple sclerosis. Folia Morphol..

[8] Giovannoni G., Comi G., Cook S., Rammohan K., Rieckmann P., Soelberg Sørensen P., Vermersch P., Chang P., Hamlett A., Musch B. (2010). A placebo-controlled trial of oral cladribine for relapsing multiple sclerosis. N. Engl. J. Med..

[9] Giovannoni G., Soelberg Sorensen P., Cook S., Rammohan K., Rieckmann P., Comi G., Dangond F., Adeniji A.K., Vermersch P. (2018). Safety and efficacy of cladribine tablets in patients with relapsing-remitting multiple sclerosis: Results from the randomized extension trial of the CLARITY study. Mult. Scler..

[10] Oh J., Walker B., Giovannoni G., Jack D., Dangond F., Nolting A., Aldridge J., Lebson L.A., Leist T.P. (2021). Treatment-emergent adverse events occurring early in the treatment course of cladribine tablets in two phase 3 trials in multiple sclerosis. Mult. Scler. J. Exp. Transl. Clin..

[11] European Medicines Agency (EMA) (2022). Meeting Highlights from the Pharmacovigilance Risk Assessment Committee (PRAC) 10–13 January 2022 [Internet]. https://www.ema.europa.eu/en/news/meeting-highlights-pharmacovigilance-risk-assessment-committee-prac-10-13-january-2022.

[12] Harbo H.F., Gold R., Tintoré M. (2013). Sex and gender issues in multiple sclerosis. Ther. Adv. Neurol. Disord..

[13] Rademaker M. (2001). Do women have more adverse drug reactions?. Am. J. Clin. Dermatol..

[14] Rydberg D.M., Mejyr S., Loikas D., Schenck-Gustafsson K., von Euler M., Malmström R.E. (2018). Sex differences in spontaneous reports on adverse drug events for common antihypertensive drugs. Eur. J. Clin. Pharmacol..

[15] Scavone C., Di Mauro C., Ruggiero R., Bernardi F.F., Trama U., Aiezza M.L., Rafaniello C., Capuano A. (2020). Severe Cutaneous Adverse Drug Reactions Associated with Allopurinol: An Analysis of Spontaneous Reporting System in Southern Italy. Drugs—Real World Outcomes.

[16] Sinagra E., Raimondo D., Cottone S., Guddo F., Gabriele Rizzo A., Amvrosiadis G., Perricone G., Cottone M., Madonia S. (2014). Does glatiramer acetate provoke hepatitis in multiple sclerosis?. Mult. Scler. Relat. Disord..

[17] Yamazaki Y., Suzuki A., Hirayanagi K., Tsukagoshi Y., Uehara R., Horiguchi K., Ohyama T., Tomaru T., Horiguchi N., Nobusawa S. (2017). An Autopsy Case of Fulminant Hepatitis in a Patient with Multiple Sclerosis Treated by Interferon-Beta-1a. Intern. Med..

[18] Nicolini L.A., Canepa P., Caligiuri P., Mikulska M., Novi G., Viscoli C., Uccelli A. (2019). Fulminant Hepatitis Associated with Echovirus 25 during Treatment with Ocrelizumab for Multiple Sclerosis. JAMA Neurol..

[19] Rigopoulou E.I., Gyftaki S., Arvaniti P., Tsimourtou V., Koukoulis G.K., Hadjigeorgiou G., Dalekos G.N. (2019). Autoimmune hepatitis in patients with multiple sclerosis: The role of immunomodulatory treatment. Clin. Res. Hepatol. Gastroenterol..

[20] Mascolo A., Scavone C., Ferrajolo C., Rafaniello C., Danesi R., Del Re M., Russo A., Coscioni E., Rossi F., Alfano R. (2021). Immune Checkpoint Inhibitors and Cardiotoxicity: An Analysis of Spontaneous Reports in Eudravigilance. Drug Saf..

[21] di Mauro G., Zinzi A., Scavone C., Mascolo A., Gaio M., Sportiello L., Ferrajolo C., Rafaniello C., Rossi F., Capuano A. (2021). PCSK9 Inhibitors and Neurocognitive Adverse Drug Reactions: Analysis of Individual Case Safety Reports from the Eudravigilance Database. Drug Saf..

[22] Rafaniello C., Ferrajolo C., Sullo M.G., Gaio M., Zinzi A., Scavone C., Gargano F., Coscioni E., Rossi F., Capuano A. (2021). Cardiac Events Potentially Associated to Remdesivir: An Analysis from the European Spontaneous Adverse Event Reporting System. Pharmaceuticals.

[23] Scavone C., Di Mauro C., Brusco S., Bertini M., di Mauro G., Rafaniello C., Sportiello L., Rossi F., Capuano A. (2019). Surveillance of adverse events following immunization related to human papillomavirus vaccines: 12 years of vaccinovigilance in Southern Italy. Expert Opin. Drug Saf..

[24] Güner M.D., Ekmekci P.E. (2019). Healthcare professionals’ pharmacovigilance knowledge and adverse drug reaction reporting behavior and factors determining the reporting rates. J. Drug Assess..

[25] Santosh K.C., Tragulpiankit P., Gorsanan S., Edwards I.R. (2013). Attitudes among healthcare professionals to the reporting of adverse drug reactions in Nepal. BMC Pharmacol. Toxicol..

[26] Kassa Alemu B., Biru T.T. (2019). Health Care Professionals’ Knowledge, Attitude, and Practice towards Adverse Drug Reaction Reporting and Associated Factors at Selected Public Hospitals in Northeast Ethiopia: A Cross-Sectional Study. BioMed Res. Int..

[27] Andrade R.J., Chalasani N., Björnsson E.S., Suzuki A., Kullak-Ublick G.A., Watkins P.B., Devarbhavi H., Merz M., Lucena M.I., Kaplowitz N. (2019). Drug-induced liver injury. Nat. Rev. Dis. Primers.

[28] Ferrajolo C., Scavone C., Donati M., Bortolami O., Stoppa G., Motola D., Vannacci A., Mugelli A., Leone R., Capuano A. (2018). Antidepressant-Induced Acute Liver Injury: A Case-Control Study in an Italian Inpatient Population. Drug Saf..

[29] Onakpoya I.J., Heneghan C.J., Aronson J.K. (2016). Post-marketing withdrawal of 462 medicinal products because of adverse drug reactions: A systematic review of the world literature. BMC Med..

[30] Saraceno L., Pirro F., Stigliano R., Agostoni E.C., Protti A. (2022). Acute idiosyncratic liver injury after Cladribine treatment for multiple sclerosis: First case report and review on associated hepatic disorders. Mult. Scler..

[31] Cook S., Vermersch P., Comi G., Giovannoni G., Rammohan K., Rieckmann P., Sørensen P.S., Hamlett A., Miret M., Weiner J. (2011). Safety and tolerability of cladribine tablets in multiple sclerosis: The CLARITY (CLAdRIbine Tablets treating multiple sclerosis orallY) study. Mult. Scler..

[32] Leist T.P., Comi G., Cree B.A., Coyle P.K., Freedman M.S., Hartung H.P., Vermersch P., Casset-Semanaz F., Scaramozza M. (2014). Effect of oral cladribine for early MS (ORACLE MS) Study Group Effect of oral cladribine on time to conversion to clinically definite multiple sclerosis in patients with a first demyelinating event (ORACLE MS): A phase 3 randomised trial. Lancet Neurol..

[33] Leist T., Cook S., Comi G., Montalban X., Giovannoni G., Nolting A., Damian D., Syed S., Galazka A. (2020). Long-term safety data from the cladribine tablets clinical development program in multiple sclerosis. Mult. Scler. Relat. Disord..

[34] Busuttil D.P., Chasty R.C., Fraser M., Copplestone J.A., Prentice A.G. (1996). Delayed reactivation of hepatitis B infection after cladribine. Lancet.

[35] Leist T., Giovannoni G., Jack D., Galazka A., Nolting A. (2023). Updated Post-Approval Safety of Cladribine Tablets in the Treatment of Multiple Sclerosis, with Particular Reference to Liver Safety (P7-3.010). Neurology.

[36] Montalban X., Leist T.P., Cohen B.A., Moses H., Campbell J., Hicking C., Dangond F. (2018). Cladribine tablets added to IFN-β in active relapsing MS: The ONWARD study. Neurol.-Neuroimmunol. Neuroinflamm..

[37] Juliusson G., Lenkei R., Tjønnfjord G., Heldal D., Liliemark J. (1995). Neutropenic fever following cladribine therapy for symptomatic hairy-cell leukemia: Predictive factors and effects of granulocyte-macrophage colony-stimulating factor. Ann. Oncol. Off. J. Eur. Soc. Med. Oncol..

[38] Comi G., Cook S., Giovannoni G., Rieckmann P., Sørensen P.S., Vermersch P., Galazka A., Nolting A., Hicking C., Dangond F. (2019). Effect of cladribine tablets on lymphocyte reduction and repopulation dynamics in patients with relapsing multiple sclerosis. Mult. Scler. Relat. Disord..

[39] Cook S., Leist T., Comi G., Montalban X., Giovannoni G., Nolting A., Hicking C., Galazka A., Sylvester E. (2019). Safety of cladribine tablets in the treatment of patients with multiple sclerosis: An integrated analysis. Mult. Scler. Relat. Disord..

[40] Deeks E.D. (2018). Cladribine Tablets: A Review in Relapsing, M.S. CNS Drugs.

[41] Rolfes L., Pfeuffer S., Huntemann N., Schmidt M., Su C., Skuljec J., Aslan D., Hackert J., Kleinschnitz K., Hagenacker T. (2022). Immunological consequences of cladribine treatment in multiple sclerosis: A real-world study. Mult. Scler. Relat. Disord..

[42] Fox R.J., Miller D.H., Phillips J.T., Hutchinson M., Havrdova E., Kita M., Yang M., Raghupathi K., Novas M., Sweetser M.T. (2012). Placebo-controlled phase 3 study of oral BG-12 or glatiramer in multiple sclerosis. N. Engl. J. Med..

[43] Gold R., Kappos L., Arnold D.L., Bar-Or A., Giovannoni G., Selmaj K., Tornatore C., Sweetser M.T., Yang M., Sheikh S.I. (2012). Placebo-controlled phase 3 study of oral BG-12 for relapsing multiple sclerosis. N. Engl. J. Med..

[44] Saida T., Yamamura T., Kondo T., Yun J., Yang M., Li J., Mahadavan L., Zhu B., Sheikh S.I. (2019). A randomized placebo-controlled trial of delayed-release dimethyl fumarate in patients with relapsing-remitting multiple sclerosis from East Asia and other countries. BMC Neurol..

[45] Gold R., Arnold D.L., Bar-Or A., Hutchinson M., Kappos L., Havrdova E., MacManus D.G., Yousry T.A., Pozzilli C., Selmaj K. (2017). Long-term effects of delayed-release dimethyl fumarate in multiple sclerosis: Interim analysis of ENDORSE, a randomized extension study. Mult. Scler..

[46] Cohen J.A., Coles A.J., Arnold D.L., Confavreux C., Fox E.J., Hartung H.P., Havrdova E., Selmaj K.W., Weiner H.L., Fisher E. (2012). Alemtuzumab versus interferon beta 1a as first-line treatment for patients with relapsing-remitting multiple sclerosis: A randomised controlled phase 3 trial. Lancet.

[47] Coles A.J., Twyman C.L., Arnold D.L., Cohen J.A., Confavreux C., Fox E.J., Hartung H.P., Havrdova E., Selmaj K.W., Weiner H.L. (2012). Alemtuzumab for patients with relapsing multiple sclerosis after disease-modifying therapy: A randomised controlled phase 3 trial. Lancet.

[48] Antonazzo I.C., Poluzzi E., Forcesi E., Riise T., Bjornevik K., Baldin E., Muratori L., De Ponti F., Raschi E. (2019). Liver injury with drugs used for multiple sclerosis: A contemporary analysis of the FDA Adverse Event Reporting System. Mult. Scler..

[49] Francis G.S., Grumser Y., Alteri E., Micaleff A., O’Brien F., Alsop J., Stam Moraga M., Kaplowitz N. (2003). Hepatic reactions during treatment of multiple sclerosis with interferon-beta-1a: Incidence and clinical significance. Drug Saf..

[50] Meunier L., Larrey D. (2023). Hepatotoxicity of Drugs Used in Multiple Sclerosis, Diagnostic Challenge, and the Role of HLA Genotype Susceptibility. Int. J. Mol. Sci..

[51] de Seze J., Canva-Delcambre V., Fajardy I., Delalande S., Stojkovic T., Godet E., Vermersch P. (2005). Autoimmune hepatitis and multiple sclerosis: A coincidental association?. Mult. Scler..

[52] Clavelou P., Castelnovo G., Pourcher V., De Sèze J., Vermersch P., Ben-Amor A.F., Savarin C., Defer G. (2023). Expert Narrative Review of the Safety of Cladribine Tablets for the Management of Relapsing Multiple Sclerosis. Neurol. Ther..

[53] EMA—Mavenclad (Cladribine)—Risk of Serious Liver Injury and New Recommendations about Liver Function Monitoring. https://www.ema.europa.eu/en/documents/dhpc/direct-healthcare-professional-communication-dhpc-mavenclad-cladribine-risk-serious-liver-injury-new_en.pdf.

[54] Postigo R., Brosch S., Slattery J., van Haren A., Dogné J.M., Kurz X., Candore G., Domergue F., Arlett P. (2018). EudraVigilance Medicines Safety Database: Publicly Accessible Data for Research and Public Health Protection. Drug Saf..

[55] European Medicines Agency (EMA) Serious Adverse Reaction [Internet]. https://www.ema.europa.eu/en/glossary/serious-adverse-reaction.

